# Effect of atherogenic index of plasma and triglyceride-glucose index on early neurological deterioration of patients with large artery atherosclerotic ischemic stroke

**DOI:** 10.1186/s13098-025-01684-x

**Published:** 2025-04-09

**Authors:** Ki-Woong Nam, Hyung-Min Kwon, Yong-Seok Lee

**Affiliations:** 1 Department of Neurology, Seoul National University College of Medicine, Seoul, Korea 101 Daehak-ro, Jongno-gu, 03080; 2 Department of Neurology, Seoul Metropolitan Government-Seoul National University Boramae Medical Center, 20 Boramae-ro 5-gil, Dongjak-gu, 07061 Seoul, Korea

**Keywords:** Large artery atherosclerotic stroke, Atherogenic index of plasma, Triglyceride-glucose index, Dyslipidemia, Prognosis, Early neurological deterioration

## Abstract

**Background:**

Stroke due to large artery atherosclerosis (LAA-stroke) has a poor early prognosis despite appropriate medical treatment. Recently, various parameters reflecting pathological conditions such as insulin resistance or atherogenic dyslipidemia have been proposed using triglyceride (TG) and other biomarkers. In this study, we evaluated the association between TG andTG-related parameters and early neurological deterioration (END) in patients with acute LAA stroke.

**Methods:**

We evaluated consecutive patients with acute LAA-stroke between January 2010 and December 2020. TG-related parameters were calculated using the following formulas: the atherogenic index of plasma (AIP) = log_10_ (TG level/high-density lipoprotein level) and TG-glucose (TyG) index = Ln (TG level x glucose level/2). END was defined as an increase of ≥ 2 in the total National Institutes of Health Stroke Scale (NIHSS) score or ≥ 1 in the motor NIHSS score within the first 72 h of admission.

**Results:**

Six hundred and forty patients with acute LAA-stroke were evaluated. In multivariable analyses, AIP (adjusted odds ratio [aOR]: 1.93, 95% confidence interval: 1.32–2.82) was closely associated with END after adjustment for confounders. The TyG index (aOR: 2.22, 95% confidence interval: 1.51–3.27) also showed close association with END. The AIP and TyG index showed significant differences between the END and no END groups only in patients with LAA-stroke caused by intracranial atherosclerosis. In addition, AIP and TyG index were closely related to END only in patients with LAA-stroke caused by artery-to-artery embolism and branch atheromatous disease mechanisms.

**Conclusions:**

We found that TG and TG-related parameters were associated with the occurrence of END in patients with acute LAA-stroke. This association appeared differently depending on the location or mechanism of the relevant vessel that caused LAA-stroke.

**Supplementary Information:**

The online version contains supplementary material available at 10.1186/s13098-025-01684-x.

## Background

Early neurological deterioration (END) is a neurological complication that occurs in patients with acute ischemic stroke [1, 2]. Although there are some differences depending on the definition, END is said to be found in approximately 10–30% of ischemic stroke patients [1]. Because END leaves more severe neurological deficit and makes worse long-term prognosis, high-risk patients should be classified and treated appropriately [2–4]. The prevalence and mechanism (e.g., recurrence, progression, hemorrhagic transformation, edema) of END are thought to vary depending on the stroke subtypes [2, 5]. And, stroke caused by large-artery atherosclerosis (LAA-stroke) has a higher frequency of END compared to stroke caused by other mechanisms [5, 6]. In particular, in the case of stroke due to intracranial atherosclerosis (ICAS), recurrence and progression occur at a high frequency even with optimal medical treatment, and intervention options (e.g., percutaneous angioplasty, stenting) cannot be considered as in stroke due to extracranial atherosclerosis (ECAS) [7–13]. Therefore, it is important to infer the pathological mechanism of END in these patient groups and establish appropriate prevention strategies in high-risk patients [14]. 

Hypercholesterolemia is a common risk factor for atherosclerosis and ischemic stroke and requires particularly strict management in patients with LAA-stroke [15]. Based on the results of previous randomized controlled studies, AHA/ACC presented a guideline that low-density lipoprotein (LDL) cholesterol should be lowered to 70 mg/dL or less, while the 2019 ESC/EAS guideline goes one step further by stating that LDL cholesterol should be lowered to 55 mg/dL for very high-risk groups [15, 16]. However, the results of several large epidemiological studies show that even if LDL cholesterol is lowered to the therapeutic range, a residual cardiovascular risk still remains [17]. It is believed that triglyceride (TG) plays a significant role in this unresolved risk. TG is a risk factor for ischemic stroke independent of LDL cholesterol, and is a modifiable risk factor that can be controlled by fibrates [9, 17–19]. However, unlike LDL cholesterol, TG has limitations in its use in clinical areas because its measurement values ​​are highly variable. Recently, TG-related parameters reflecting various pathological conditions that may affect cerebrovascular disease were proposed using TGs and other biomarkers [20, 21]. Representative examples include the atherogenic index of plasma (AIP), which reflects atherogenic dyslipidemia, and the triglyceride-glucose (TyG) index, which reflects insulin resistance [20, 21]. These values ​​represent relatively stable measured values ​​because the log function is used in the calculation process.

In this study, we aimed to investigate the association between TG and TG-related parameters and early neurological deterioration (END) in patients with acute LAA-stroke. In addition, we compared the clinical significance of these parameters according to the location of symptomatic atherosclerotic lesions (e.g., ICAS and ECAS) and the LAA-stroke mechanism.

## Methods

### Study population

From the prospectively collected acute stroke registry of Seoul Metropolitan Government-Seoul National University Boramae Medical Center (SMG-SNUBMC), we retrospectively enrolled patients with acute ischemic stroke due to the LAA mechanism between January 2010 and December 2020. Classification of LAA-stroke was based on the Trial of Org 10,172 in Acute Stroke Treatment classification [22]. All patients with acute ischemic stroke admitted to our center underwent brain magnetic resonance imaging (MRI), magnetic resonance arteriography (MRA), echocardiography, electrocardiography, and laboratory examination for stroke etiological evaluation. Among these, patients were excluded according to the following exclusion criteria: (1) arrival more than 72 h after onset of symptoms, (2) receiving intravenous thrombolysis or intraarterial thrombectomy, and (3) missing data on major covariates. Finally, 640 patients with acute LAA-stroke were included in the analysis.

This retrospective cross-sectional study was approved by the Institutional Review Board (IRB) of SMG-SNUBMC (IRB number: 30-2022-105). The IRB waived the requirement of informed consent due to the retrospective design and use of anonymized information.

### Demographic, clinical, laboratory, and radiological variables

We evaluated demographic and clinical factors, including age, sex, symptom onset to door time, hypertension, diabetes, dyslipidemia, ischemic heart disease, current smoking, history of stroke, initial stroke severity, use of antihypertensive agent/glucose-lowering agent/lipid-lowering agent, and systolic and diastolic blood pressure. Initial stroke severity was determined based on the National Institutes of Health Stroke Scale (NIHSS), which was measured daily by the attending physician from the time of the patient’s hospitalization, regardless of the study. All hospitalized patients underwent laboratory examination within the first 24 h of admission. These included glucose profiles (e.g., hemoglobin A1c, fasting glucose), lipid profiles (e.g., total/LDL/HDL cholesterol, and TG), white blood cell counts, and high-sensitivity C-reactive protein levels. Glucose and lipid profiles, including fasting glucose, TG, and HDL cholesterol were measured after overnight fasting for 12 h.

Brain MRI and MRA were performed on a 3.0-T MR scanner (Achieva Philips, Eindhoven, the Netherlands) for all patients within 24 h after admission. According to the location and number of lesions on the diffusion-weighted image (DWI) and the location, shape, and stenosis degree of the atherosclerotic vessel on MRA, we classified the relevant vessels that caused the index LAA-stroke into ICAS and ECAS. As our study population included patients with LAA-stroke, all patients were classified into either the ICAS or ECAS group. Additionally, considering the relationship between DWI lesions and relevant vessels, we defined four types of LAA-stroke mechanisms: artery-to-artery embolism, branch atheromatous disease, border-zone, and in situ thrombosis [23, 24]. Because the classification of relevant vessels and the LAA-stroke mechanism is performed routinely during the treatment process upon hospitalization, the medical record obtained by the attending physician was cited as the primary classification. A second classification was made independently by an investigator (K.-W.N.), and any discrepancies between the results were finalized through consultation with the third raters (H.-M.K. and Y.-S.L.).

### Major variables

We defined the two major variables of this study, AIP and TyG index, according to the following formulas: AIP = log10 [TG (mg/dL) / HDL cholesterol (mg/dL)] and TyG index = Ln [fasting TG (mg/dL) x fasting glucose (mg/dL)/2] [20, 21]. 

### Outcome variable

In this study, we chose END as the main outcome variable. END was defined as an increase in total NIHSS score by ≥ 2 points or motor NIHSS score by ≥ 1 point within 72 h after hospitalization [25].

### Statistical analysis

Continuous variables that followed a normal distribution are expressed as mean ± standard deviation, and variables that did not are expressed as median + interquartile range. Categorical variables are expressed as frequencies with percentages. Differences in baseline characteristics between the patient groups with and without END were compared using the Student’s t-test and Mann-Whitney U-test for continuous variables and the chi-squared test and Fisher’s exact test for categorical variables (univariate analyses). Based on the results of univariate analysis, we attempted to find possible predictors of END by introducing variables with *P* < 0.05 into a multivariable logistic regression model. However, because the AIP and TyG index share the same variables in their calculation formulas, multivariable analyses of models 1 and 2 using each of these TG-related parameters were performed separately. For the same reason, TG was not introduced into the multivariable analysis simultaneously with these TG-related parameters, and glucose was not introduced together with the TyG index.

The LAA-stroke in our study population was caused by ICAS or ECAS. Therefore, we compared the difference between AIP and TyG index values ​​according to symptomatic relevant vessels. In addition, the differences in TG-related parameter values ​​between patient groups with and without END in ICAS and ECAS, respectively, were compared. Meanwhile, since LAA-stroke can be divided into four types of mechanisms, the differences in TG-related parameter values ​​according to these mechanisms were also compared. All statistical analyses were performed using SPSS version 23.0 (IBM SPSS, Chicago, IL, USA), and all variables with *P* < 0.05 were considered statistically significant.

## Results

We evaluated 640 patients with acute LAA-stroke (mean age: 69.2 ± 12.4 years, male sex: 64.1%). The median time from symptom onset to hospital visit was 12 [4.0–30.0] hours, and the median initial NIHSS score was 3 [2–6]. Among the study population, 114 (17.8%) people experienced an END event. The mean value of AIP was 0.95 ± 0.59, and the median value of TyG index was 8.58 [8.24–9.01]. Other detailed baseline characteristics are described in Table 1.

**Table 1:** Baseline characteristics of the study population (*n* = 640)

Demographic & clinical variables	
Age, y [IQR]	71 [61–78]
Sex, male, n (%)	410 (64.1)
Symptom onset to door time, h [IQR]	12.0 [4.0–30.0]
Hypertension, n (%)	432 (67.5)
Diabetes, n (%)	214 (33.4)
Dyslipidemia, n (%)	149 (23.3)
Ischemic heart disease, n (%)	46 (7.2)
Current smoking, n (%)	209 (32.7)
History of stroke, n (%)	110 (17.2)
Initial NIHSS score [IQR]	3 [2–6]
Use of antihypertensive agent, n (%)	356 (55.6)
Use of glucose-lowering agent, n (%)	167 (26.1)
Use of lipid-lowering agent, n (%)	108 (16.9)
Systolic blood pressure, mmHg [IQR]	158 [141–180]
Diastolic blood pressure, mmHg [IQR]	85 [76–95]
**Laboratory variables**	
Hemoglobin A1c, % [IQR]	6.0 [5.6-7.0]
Fasting glucose, mg/dL [IQR]	100 [90–123]
Total cholesterol, mg/dL [SD]	189 ± 44
LDL cholesterol, mg/dL [IQR]	111 [85–136]
HDL cholesterol, mg/dL [IQR]	41 [34–49]
Triglyceride, mg/dL [IQR]	103 [78–145]
White blood cell counts, x 10^3^/uL [IQR]	7.60 [6.09–9.29]
High-sensitivity CRP, mg/dL [IQR]	0.16 [0.06–0.50]
Atherogenic index of plasma. [SD]	0.95 ± 0.59
Triglyceride-glucose index, [IQR]	8.58 [8.24–9.01]
**Radiological variables**	
LAA-stroke mechanism, (%)	
Artery-to-artery embolism	214 (33.4)
Branch atheromatous disease	232 (36.3)
Border-zone	131 (20.5)
In situ thrombosis	63 (9.8)
Relevant vessel, n (%)	
Intracranial atherosclerosis	442 (69.1)
Extracranial atherosclerosis	198 (30.9)
**Outcome variables**	
Early neurological deterioration, n (%)	114 (17.8)

LAA = large artery atherosclerosis, NIHSS = National Institutes of Health Stroke Scale, LDL = low-density lipoprotein, HDL = high-density lipoprotein, CRP = c-reactive protein

In a univariate analysis comparing baseline characteristics between patients who experienced END and those who did not, the END group was associated with age, symptom onset to door time, LAA-stroke mechanism, relevant vessel, initial NIHSS score, fasting glucose and TG levels, AIP, and TyG index (Table 2).

**Table 2:** Comparisons of baseline characteristics of patients with and without early neurological deterioration

	No END (*n* = 556)	END (*n* = 126)	*P* value
Age, y [IQR]	70 [61–77]	74 [62–82]	0.020
Sex, male, n (%)	345 (65.6)	65 (57.0)	0.084
Symptom onset to door time, h [IQR]	13.0 [4.5–31.0]	9.0 [3.0–24.0]	0.022
Hypertension, n (%)	351 (66.7)	81 (71.1)	0.372
Diabetes, n (%)	176 (33.5)	38 (33.3)	0.979
Dyslipidemia, n (%)	122 (23.2)	27 (23.7)	0.911
Ischemic heart disease, n (%)	39 (7.4)	7 (6.1)	0.633
Current smoking, n (%)	177 (33.7)	32 (28.1)	0.249
History of stroke, n (%)	92 (17.5)	18 (15.8)	0.663
Use of antihypertensive agent, n (%)	289 (54.9)	67 (58.8)	0.456
Use of glucose-lowering agent, n (%)	140 (26.6)	27 (23.7)	0.518
Use of lipid-lowering agent, n (%)	89 (16.9)	19 (16.7)	0.948
Systolic blood pressure, mmHg [IQR]	157 [140–180]	163 [144–184]	0.116
Diastolic blood pressure, mmHg [IQR]	85 [76–95]	85 [77–94]	0.982
LAA-stroke mechanism, n (%)			0.001
Artery-to-artery embolism	189 (35.9)	25 (21.9)	0.004
Branch atheromatous disease	182 (34.6)	50 (43.9)	0.062
Border-zone	112 (21.3)	19 (16.7)	0.267
In situ thrombosis	43 (8.2)	20 (17.5)	0.002
Relevant vessel, n (%)			0.038
Intracranial atherosclerosis	354 (67.3)	88 (77.2)	
Extracranial atherosclerosis	172 (32.7)	26 (22.8)	
Initial NIHSS score [IQR]	3 [1–6]	5 [3–9]	< 0.001
Hemoglobin A1c, % [IQR]	6.0 [5.6–6.9]	6.1 [5.7-7.0]	0.454
Fasting glucose, mg/dL [IQR]	99 [89–120]	105 [94–134]	0.004
Total cholesterol, mg/dL [SD]	188 ± 42	193 ± 50	0.289
LDL cholesterol, mg/dL [SD]	112 ± 37	114 ± 42	0.640
HDL cholesterol, mg/dL [IQR]	41 [34–49]	41 [34–47]	0.347
Triglyceride, mg/dL [IQR]	100 [76–137]	116 [86–164]	0.004
White blood cell, x 10^3^/uL [IQR]	7.52 [6.05–9.24]	8.02 [6.09–9.62]	0.137
High-sensitivity CRP, mg/dL [IQR]	0.16 [0.06–0.50]	0.21 [0.07–0.50]	0.122
AIP, [SD]	0.92 ± 0.58	1.09 ± 0.58	0.005
TyG index, [IQR]	8.54 [8.22–8.95]	8.86 [8.40–9.17]	< 0.001

END = early neurological deterioration, LAA = large artery atherosclerosis, NIHSS = National Institutes of Health Stroke Scale, LDL = low-density lipoprotein, HDL = high-density lipoprotein, CRP = c-reactive protein, AIP = atherogenic index of plasma, TyG index = triglyceride-glucose index

In multivariable logistic regression analysis, AIP was closely associated with END after adjustment for confounders (adjusted odds ratio [aOR]: 1.93, 95% confidence interval [CI]: 1.32–2.82, model 1). Age, branch atheromatous disease, in situ thrombosis, and initial NIHSS score were also closely related to END, regardless of the AIP. In another multivariable analysis model using different confounders, the TyG index showed a close association with END (aOR: 2.22, 95% CI: 1.51–3.27, model 2, Table 3). In addition, even when analyzing END that occurred within 24 h as an outcome, TyG index and AIP still showed a statistically significant relationship (additional file 1: Table S1). Also in a multivariable logistic regression analysis that analyzed TG itself as an independent variable, TG showed a statistically significant association with END (Additional file 2: Table S2). When comparing predictive ability using receiver operating characteristic (ROC) curves, the TyG index showed a slightly higher area under the curve value than AIP and TG (Additional File 3: Figure S1).

**Table 3:** Multivariable logistic regression analysis of possible predictors of early neurological deterioration

	Crude OR (95% CI)	*P*-value	Adjusted OR (95% CI)	*P*-value
**Atherogenic index of plasma**				
Age	1.02 [1.00-1.04]	0.035	1.02 [1.00-1.04]	0.040
Symptom onset to door time	0.99 [0.98-1.00]	0.122	0.99 [0.98-1.00]	0.144
LAA-stroke mechanism		0.001		0.005
Artery-to-artery embolism	Ref	Ref	Ref	Ref
Branch atheromatous disease	2.08 [1.23–3.50]	0.006	2.34 [1.35–4.04]	0.002
Border-zone	1.28 [0.68–2.43]	0.447	1.32 [0.68–2.57]	0.408
In situ thrombosis	3.52 [1.79–6.91]	< 0.001	2.66 [1.28–5.52]	0.009
Initial NIHSS score	1.08 [1.05–1.12]	< 0.001	1.08 [1.04–1.12]	< 0.001
Fasting glucose	1.01 [1.00-1.01]	0.021	1.00 [1.00-1.01]	0.112
Atherogenic index of plasma	1.66 [1.17–2.36]	0.005	1.93 [1.32–2.82]	0.001
**Triglyceride-glucose index**				
Age	1.02 [1.00-1.04]	0.035	1.02 [1.00-1.04]	0.029
Symptom onset to door time	0.99 [0.98-1.00]	0.122	0.99 [0.98-1.00]	0.161
LAA-stroke mechanism		0.001		0.005
Artery-to-artery embolism	Ref	Ref	Ref	Ref
Branch atheromatous disease	2.08 [1.23–3.50]	0.006	2.33 [1.35–4.03]	0.002
Border-zone	1.28 [0.68–2.43]	0.447	1.34 [0.69–2.60]	0.395
In situ thrombosis	3.52 [1.79–6.91]	< 0.001	2.75 [1.32–5.73]	0.007
Initial NIHSS score	1.08 [1.05–1.12]	< 0.001	1.08 [1.04–1.12]	< 0.001
Triglyceride-glucose index	1.80 [1.27–2.54]	0.001	2.22 [1.51–3.27]	< 0.001

OR = odds ratio, LAA = large artery atherosclerosis, NIHSS = National Institutes of Health Stroke Scale

**Fig. 1:**
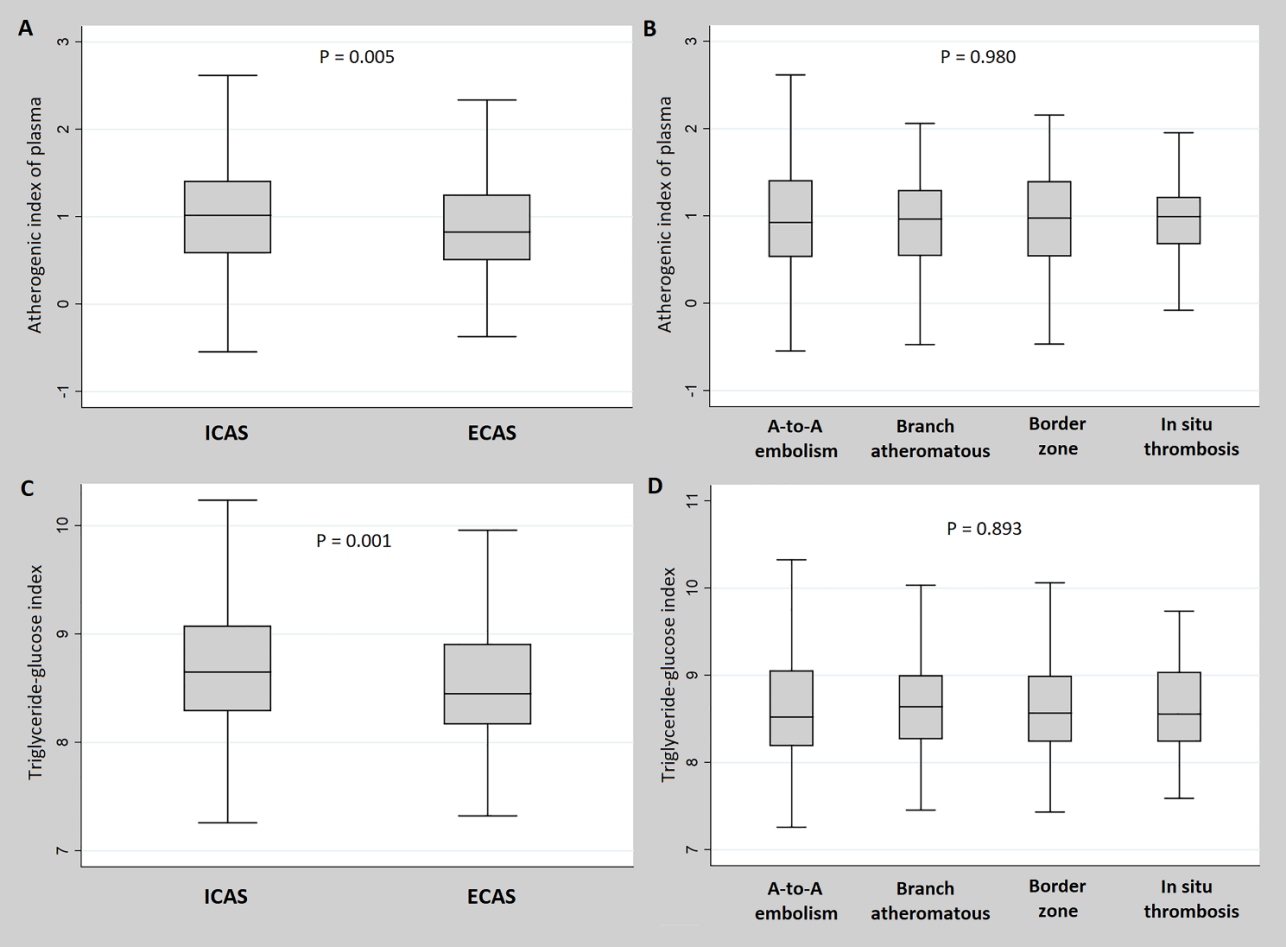
Comparison of triglyceride-related parameters according to relevant vessel and large artery atherosclerosis stroke mechanisms. ICAS = intracranial atherosclerosis, ECAS = extracranial atherosclerosis, A-to-A = artery-to-artery, AIP = atherogenic index of plasma, TyG index = triglyceride-glucose index, LAA = large artery atherosclerosis. Both AIP (*P* = 0.005) and TyG index (*P* = 0.001) were higher in LAA-stroke by ICAS than in that by ECAS (**A**, **C**). However, both AIP (*P* = 0.980) and TyG index (*P* = 0.893) showed no difference depending on LAA-stroke mechanism (**B**, **D**)

**Fig. 2:**
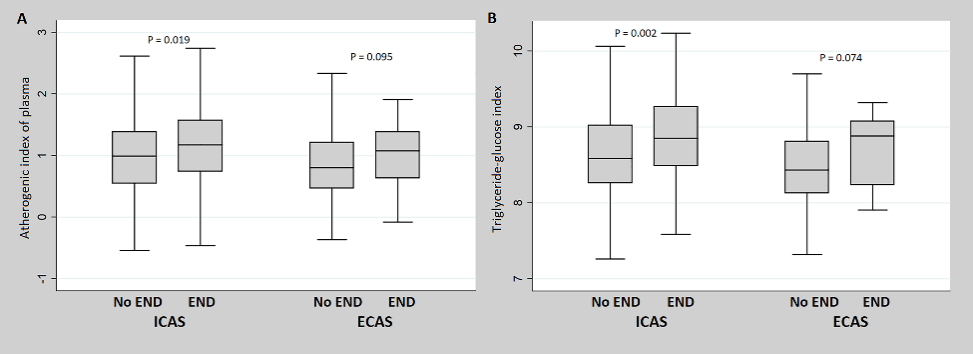
Comparison of differences in triglyceride-related parameters according to END status for each relevant vessel. END = early neurological deterioration, ICAS = intracranial atherosclerosis, ECAS = extracranial atherosclerosis, AIP = atherogenic index of plasma, TyG index = triglyceride-glucose index, LAA = large artery atherosclerosis. In patients with LAA-stroke caused by ICAS, a statistically significant difference in AIP (*P* = 0.019) and TyG index (*P* = 0.002) values ​​was observed between the END and no END groups. However, in patients with LAA-stroke caused by ECAS, AIP (*P* = 0.095) and TyG index (*P* = 0.074) were not associated with END

**Fig. 3:**
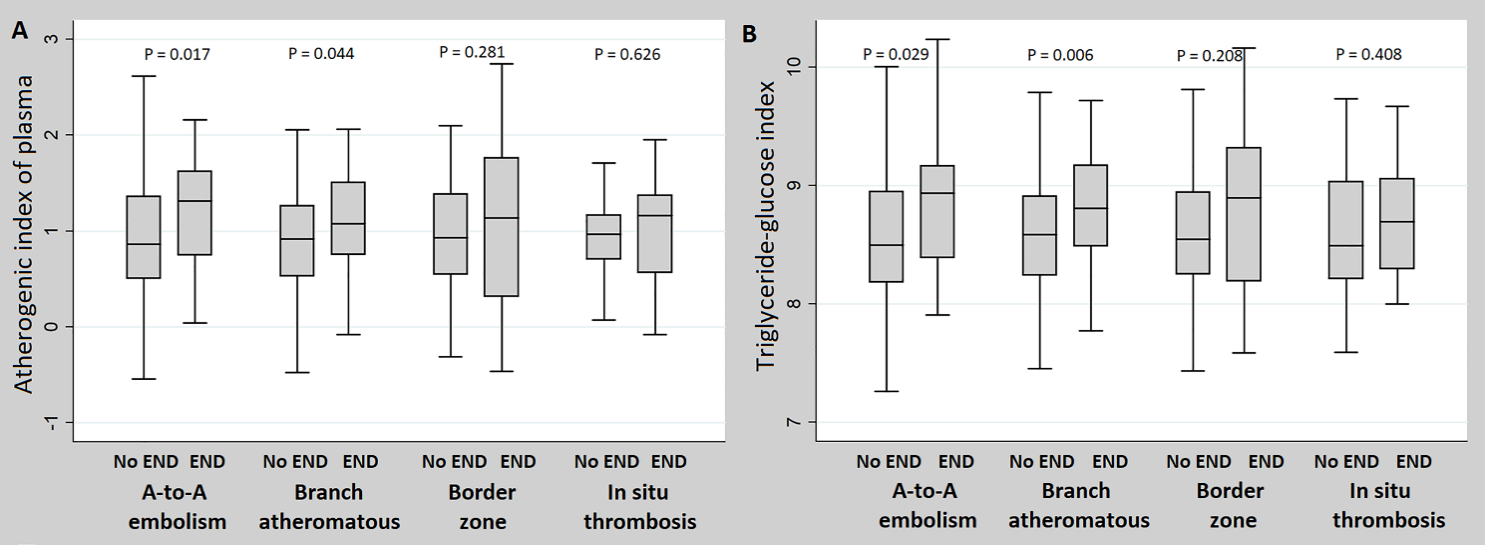
Comparison of differences in triglyceride-related parameters according to END by the large artery atherosclerosis stroke mechanism. END = early neurological deterioration, A-to-A = artery-to-artery, AIP = atherogenic index of plasma, TyG index = triglyceride-glucose index, LAA = large artery atherosclerosis. When compared according to the LAA-stroke mechanism, both AIP and TyG index showed statistically significant differences between the END and no END groups only in the artery-to-artery embolism and branch atheromatous disease mechanisms

The AIP (*P* = 0.005) and TyG index (*P* = 0.001) were statistically significantly higher in patients with LAA-stroke caused by ICAS than by ECAS. However, neither the AIP nor TyG index showed statistically significant differences between the four types of LAA-stroke mechanisms (Fig. 1). Additionally, in patients with LAA-stroke caused by ICAS, the AIP (*P* = 0.019) and TyG index (*P* = 0.002) showed significant differences between the groups that did and did not experience END. However, in patients with LAA-stroke caused by ECAS, only a statistical trend was observed between the two groups, with no significant difference (Fig. 2). In the comparison between the END and no END groups according to the LAA-stroke mechanism, TyG index and AIP values ​​showed statistically significant differences only in patients with artery-to-artery embolism and branch atheromatous disease mechanism (Fig. 3).

## Discussion

In this study, we found that TG and TG-related parameters were associated with the occurrence of END in patients with acute LAA-stroke. This close association was more frequently observed in LAA-stroke patients caused by ICAS. Additionally, the influence of AIP and TyG index appeared in different ways depending on the LAA-stroke mechanism.

As mentioned earlier, hypertriglyceridemia itself is one of the risk factors for ischemic stroke, especially LAA-stroke [18, 19]. The practice guideline for symptomatic ICAS patients presented by the American Academy of Neurology in 2022 suggests that TG is a stronger risk factor than LDL cholesterol in this patient group [10]. TG-rich lipoproteins, such as very low-density lipoprotein or chylomicron, are so small that they can enter the arterial intima [9, 17]. Substances that enter the arterial wall are broken down by lipoprotein lipase and liberated in the form of free fatty acid [9, 17]. This can cause low-grade inflammation, foam cell formation, and progression or rupture of atherosclerotic plaque [26, 27]. In fact, in a previous study conducted on patients with atherothrombotic stroke, hypertriglyceridemia significantly increased the risk of developing further vascular events in the first month [9]. However, since then, the influence of hypertriglyceridemia on stroke recurrence gradually decreased over time, which suggests that high TG levels mainly act in the acute period. The AIP reflects atherogenic dyslipidemia characterized by high TG, low HDL cholesterol, and small and dense LDL cholesterol particles [28]. In our study, HDL cholesterol level, a component of AIP, did not have statistical significance in univariate analysis. Therefore, we believe that the close association between AIP and END may be due to hypertriglyceridemia rather than the overall pathological condition of atherogenic dyslipidemia.

The TyG index was presented in 2008 as a marker of insulin resistance and is considered a reliable parameter that can replace the homeostatic model assessment for insulin resistance [20, 29, 30]. Insulin resistance affects the formation and rupture of atherosclerotic plaques through various pathways. It inhibits insulin signaling at the intimal cell level, causing endothelial dysfunction, chronic inflammation, and abnormal responses to oxidative stress [30–33]. Additionally, it promotes the formation of foam cells, aiding vulnerable plaque formation and inducing plaque necrosis [31, 33, 34]. The TyG index, which simultaneously reflects hepatic and peripheral insulin resistance, has shown a close association with various cardiovascular and cerebrovascular atherosclerotic diseases [29, 32, 34]. Our previous study also showed that the TyG index was closely correlated with early radiological recurrence in patients with acute stroke, and interestingly this association was found only in patients with LAA-stroke [35]. 

TG and TG-related parameters had higher values in patients with LAA-stroke caused by ICAS than by ECAS and showed a statistically significant association with END only in patients with ICAS. We believe that this is due to differences in vessel wall structure and metabolism between ICAS and ECAS [17, 36]. ICAS has thinner media, less adventitia, and only a few elastic fibers compared to ECAS [17, 37]. This causes differences in molecular permeability, which can also cause differences in damage due to the entry of TG-rich lipoprotein. Additionally, in terms of metabolism, intracranial arteries have higher antioxidant enzyme activity than extracranial arteries [17, 37]. Therefore, ICAS may be more vulnerable than ECAS in an environment of antioxidant depletion, such as insulin resistance, and may cause atherogenesis and plaque rupture through endothelial dysfunction [36]. This is proven in the fact that in previous studies, ICAS, with or without symptoms, showed a closer relationship with various metabolic diseases or insulin resistance than ECAS [33, 36, 38]. 

Although they are grouped together and classified, LAA-stroke has relatively diverse pathological mechanisms compared to strokes caused by other mechanisms [7, 24]. In our data, TG and TG-related parameters seemed to show significant differences between the END and no END groups only in patients with artery-to-artery embolism and branch atheromatous disease. This suggests that these TG-rich lipoproteins or insulin resistance is mainly involved in plaque vulnerability in the acute period, acting as an embolic focus through rupture or blocking of the perforating artery during the healing process after silent rupture. On the other hand, TG-related parameters do not seem to play a significant role in in situ thrombosis formation due to distal hypoperfusion or intravascular thrombosis formation in vessels already suffering from severe stenosis. However, when interpreting these results, the potential bias due to small sample sizes in the subgroups must be considered.

There are several limitations in interpreting the results of this study: First, this study is a retrospective cross-sectional study. Association exists between TG-related parameters and END, but this does not imply a causal relationship. Second, we conducted our analysis using a single value measured at the time of hospitalization. As we defined END as an event that occurs within 72 h of hospitalization, it would have been more helpful to prove the causal relationship if TG, AIP, and TyG index values ​​were measured multiple times and the occurrence of END could be observed according to the change. Third, this study was conducted only on Asians, especially Koreans. Therefore, interpretation of results based on race or genetic differences is also necessary. Fourth, we excluded patients who underwent intravenous thrombolysis or intraarterial thrombectomy during the selection process of the study population. Although LAA-stroke patients received relatively uniform acute-phase treatment, slight differences in treatment methods between individuals should be considered when interpreting the results. Fifth, this study population is basically patients with LAA-stroke, which is thought to have occurred due to the atherosclerosis mechanism. However, it is possible that some of these patients were incorrectly included with non-atherosclerotic vasculopathy such as vasculitis or dissection. Sixth, considering the definition of END, we included patients with acute LAA-stroke within 72 h of symptom onset. Therefore, if END occurs before admission, there is a possibility that the prevalence of END may be underestimated. Last, including other test results that could explain the pathological mechanism (e.g., follow-up MRI, micro-embolic signal on transcranial Doppler sonography, and high-resolution vessel wall image) may have provided a more accurate explanation of the mechanisms by which hypertriglyceridemia and TyG index act on END.

## Conclusion

We found that TG and TG-related parameters were closely associated with END in patients with acute LAA-stroke. Since both TG and TyG index can be easily measured with a simple blood test, it is expected that it can be measured at the time of hospitalization to easily classify high-risk groups for acute progression in LAA-stroke. In addition, as a new fibrate that reduces the side effects of existing fenofibrate has recently been developed and is undergoing phase 3 clinical trials, TG-based risk group classification and early intervention may be a new option for patients with LAA-stroke with poor initial prognosis [9, 17]. However, our results must be verified through subsequent prospective studies.

## Electronic supplementary material

Below is the link to the electronic supplementary material.

Supplementary Material 1

## Data Availability

No datasets were generated or analysed during the current study.

## Abbreviations

LAA: Large artery atherosclerosis
ICAS: Intracranial atherosclerosis
ECAS: Extracranial atherosclerosis
LDL: Low-density lipoprotein
TG: Triglyceride
AIP: Atherogenic index of plasma
TyG index: Triglyceride-glucose index
END: Early neurological deterioration
NIHSS: National Institutes of Health Stroke Scale
DWI: Diffusion-weighted imaging
