# Supplementary material for: Effect of atherogenic index of plasma and triglyceride-glucose index on early neurological deterioration of patients with large artery atherosclerotic ischemic stroke
Source: Diabetol Metab Syndr. 2025 Apr 9;17:123. doi: 10.1186/s13098-025-01684-x (PMC11980276; doi:10.1186/s13098-025-01684-x)

**Additional file 1. Multivariable logistic regression analysis of possible predictors of early neurological deterioration (<24h)**

|  | Crude OR  (95% CI) | *P*-value | Adjusted OR  (95% CI) | *P*-value |
| --- | --- | --- | --- | --- |
| **Atherogenic index of plasma** | | | | |
| Age | 1.01 [0.99-1.03] | 0.319 | 1.01 [0.99-1.03] | 0.345 |
| Symptom onset to door time | 0.99 [0.96-1.02] | 0.556 | 0.99 [0.96-1.03] | 0.621 |
| LAA mechanism |  | 0.005 |  | 0.011 |
| Artery-to-artery embolism | Ref | Ref | Ref | Ref |
| Branch atheromatous disease | 2.23 [1.24-4.00] | 0.007 | 2.50 [1.36-4.58] | 0.003 |
| Border-zone | 1.33 [0.64-2.76] | 0.445 | 1.30 [0.62-2.75] | 0.491 |
| In situ thrombosis | 3.23 [1.55-6.73] | 0.002 | 2.48 [1.13-5.40] | 0.023 |
| Initial NIHSS score | 1.07 [1.03-1.12] | < 0.001 | 1.07 [1.03-1.12] | 0.002 |
| Fasting glucose | 1.01 [1.00-1.01] | 0.135 | 1.00 [1.00-1.01] | 0.470 |
| Atherogenic index of plasma | 1.57 [1.06-2.31] | 0.023 | 1.75 [1.15-2.66] | 0.009 |
|  |  |  |  |  |
| **Triglyceride-glucose index** | | | | |
| Age | 1.01 [0.99-1.03] | 0.319 | 1.01 [0.99-1.03] | 0.300 |
| Symptom onset to door time | 0.99 [0.96-1.02] | 0.556 | 0.99 [0.96-1.03] | 0.655 |
| LAA mechanism |  | 0.005 |  | 0.010 |
| Artery-to-artery embolism | Ref | Ref | Ref | Ref |
| Branch atheromatous disease | 2.23 [1.24-4.00] | 0.007 | 2.48 [1.35-4.55] | 0.003 |
| Border-zone | 1.33 [0.64-2.76] | 0.445 | 1.31 [0.62-2.76] | 0.486 |
| In situ thrombosis | 3.23 [1.55-6.73] | 0.002 | 2.57 [1.17-5.64] | 0.018 |
| Initial NIHSS score | 1.07 [1.03-1.12] | < 0.001 | 1.07 [1.03-1.12] | 0.001 |
| Triglyceride-glucose index | 1.70 [1.15-2.53] | 0.008 | 1.94 [1.26-2.99] | 0.003 |

OR = odds ratio, LAA = large artery atherosclerosis, NIHSS = National Institutes of Health Stroke Scale

**Additional file 2. Multivariable logistic regression analysis using triglyceride**

|  | Crude OR  (95% CI) | *P*-value | Adjusted OR  (95% CI) | *P*-value |
| --- | --- | --- | --- | --- |
| **Triglyceride** | | | | |
| Age | 1.02 [1.00-1.04] | 0.035 | 1.02 [1.00-1.04] | 0.038 |
| Symptom onset to door time | 0.99 [0.98-1.00] | 0.122 | 0.99 [0.98-1.00] | 0.168 |
| LAA mechanism |  | 0.001 |  | 0.004 |
| Artery-to-artery embolism | Ref | Ref | Ref | Ref |
| Branch atheromatous disease | 2.08 [1.23-3.50] | 0.006 | 2.34 [1.36-4.04] | 0.002 |
| Border-zone | 1.28 [0.68-2.43] | 0.447 | 1.34 [0.69-2.60] | 0.389 |
| In situ thrombosis | 3.52 [1.79-6.91] | < 0.001 | 2.77 [1.34-5.75] | 0.006 |
| Initial NIHSS score | 1.08 [1.05-1.12] | < 0.001 | 1.08 [1.04-1.12] | < 0.001 |
| Fasting glucose | 1.01 [1.00-1.01] | 0.021 | 1.00 [1.00-1.01] | 0.140 |
| Triglyceride | 1.00 [1.00-1.01] | 0.035 | 1.01 [1.00-1.01] | 0.006 |

OR = odds ratio, LAA = large artery atherosclerosis, NIHSS = National Institutes of Health Stroke Scale

**Additional File 3. Receiver Operating Characteristic Curves of AIP, TyG Index, and TG for END**


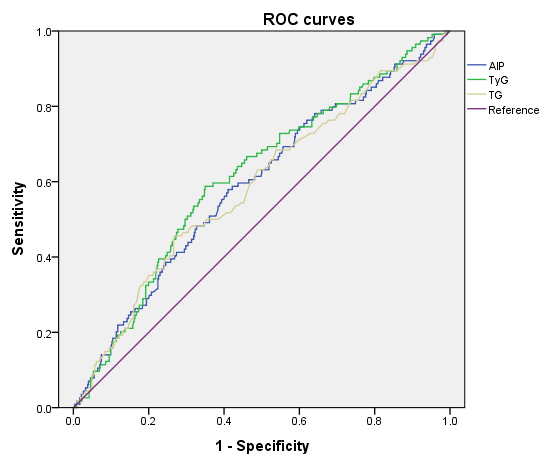

Supplement: Supplementary file 1 — Supplementary Material 1 [file 13098_2025_1684_MOESM1_ESM.docx]
